# Impact of Pregnancy-Associated Malaria on Infant Malaria Infection in Southern Benin

**DOI:** 10.1371/journal.pone.0080624

**Published:** 2013-11-13

**Authors:** Sophie Borgella, Nadine Fievet, Bich-Tram Huynh, Samad Ibitokou, Gbetognon Hounguevou, Jacqueline Affedjou, Jean-Claude Sagbo, Parfait Houngbegnon, Blaise Guezo-Mévo, Achille Massougbodji, Adrian J. F. Luty, Michel Cot, Philippe Deloron

**Affiliations:** 1 Centre d’étude et de recherche sur le paludisme associé à la grossesse et à l’enfance (CERPAGE), Faculté des Sciences de la Santé, Université d’Abomey-Calavi, Cotonou, Benin; 2 Institut de Recherche pour le Développement, UMR 216, Mère et enfant face aux infections tropicales, Paris, France; 3 PRES Sorbonne Paris Cité, Université Paris Descartes, Faculté de Pharmacie, Paris, France; London School of Hygiene and Tropical Medicine, United Kingdom

## Abstract

**Background:**

Infants of mothers with placental *Plasmodium falciparum* infections at delivery are themselves more susceptible to malaria attacks or to infection in early life.

**Methodology/ Principal Findings:**

To assess the impact of either the timing or the number of pregnancy-associated malaria (PAM) infections on the incidence of parasitemia or malaria attacks in infancy, we followed 218 mothers through pregnancy (monthly visits) up to delivery and their infants from birth to 12 months of age (fortnightly visits), collecting detailed clinical and parasitological data. After adjustment on location, mother’s age, birth season, bed net use, and placental malaria, infants born to a mother with PAM during the third trimester of pregnancy had a significantly increased risk of infection (OR [95% CI]: 4.2 [1.6; 10.5], p = 0.003) or of malaria attack (4.6 [1.7; 12.5], p = 0.003). PAM during the first and second trimesters had no such impact. Similarly significant results were found for the effect of the overall number of PAM episodes on the time to first parasitemia and first malaria attack (HR [95% CI]: 2.95 [1.58; 5.50], p = 0.001 and 3.19 [1.59; 6.38], p = 0.001) respectively.

**Conclusions/ Significance:**

This study highlights the importance of protecting newborns by preventing repeated episodes of PAM in their mothers.

## Introduction

Each year 125 million pregnancies are at risk of infection with *Plasmodium falciparum* in endemic areas [1]. Pregnancy-associated malaria (PAM) has adverse consequences for both the pregnant woman and her fetus by causing anemia and abortion, but also low birth weight (LBW), being responsible for approximately 200,000 infant deaths per year [2,3].

Parasite specific adhesion in the placental intervillous spaces [4,5] contributes to placental insufficiency [6-10]. These alterations are thought to be related, either directly or indirectly, to fetal growth restriction and premature birth, both of which lead to an increased rate of LBW, itself an important risk factor for infant mortality [10,11]. The impact of placental malaria on infant health is well documented and includes greater susceptibility to malaria and anemia in those born to mothers with a parasitized placenta [12-17]. All these studies have, nevertheless, focused on placental infection at delivery, with no exploration of the mother’s history of infection earlier during pregnancy. Our aim here was to assess the impact of PAM, taking account of both the timing of its occurrence and the number of PAM infections, on the occurrence of malaria attacks during the first year of life of the offspring. Within the framework of the STOPPAM (Strategies TO Prevent Pregnancy-Associated Malaria) project in Benin, we therefore closely followed 218 women during their pregnancy and their 218 children during their first year of life, collecting all relevant clinical and parasitological data to allow us to compile detailed and accurate infection histories for each mother-infant pair.

## Methods

### Study area

The project was conducted in the Come district (Mono Province), in Southern Benin, 70 km from Cotonou. Three dispensaries were involved: Come, a semi-rural town, Akodeha and Ouedeme Pedah, two villages situated on the banks of Lake Ahémé (Figure 1). The climate is subtropical, with two rainy seasons (April-July, October-November) and annual rainfall is >1300mm. Malaria is mesoendemic with 1 to 35 bites/person/year entomological inoculation rate [18]. The principal vectors are *Anopheles gambiae* and *An. funestus*. *Plasmodium falciparum* is the predominant species transmitted (97%). 

**Figure:**
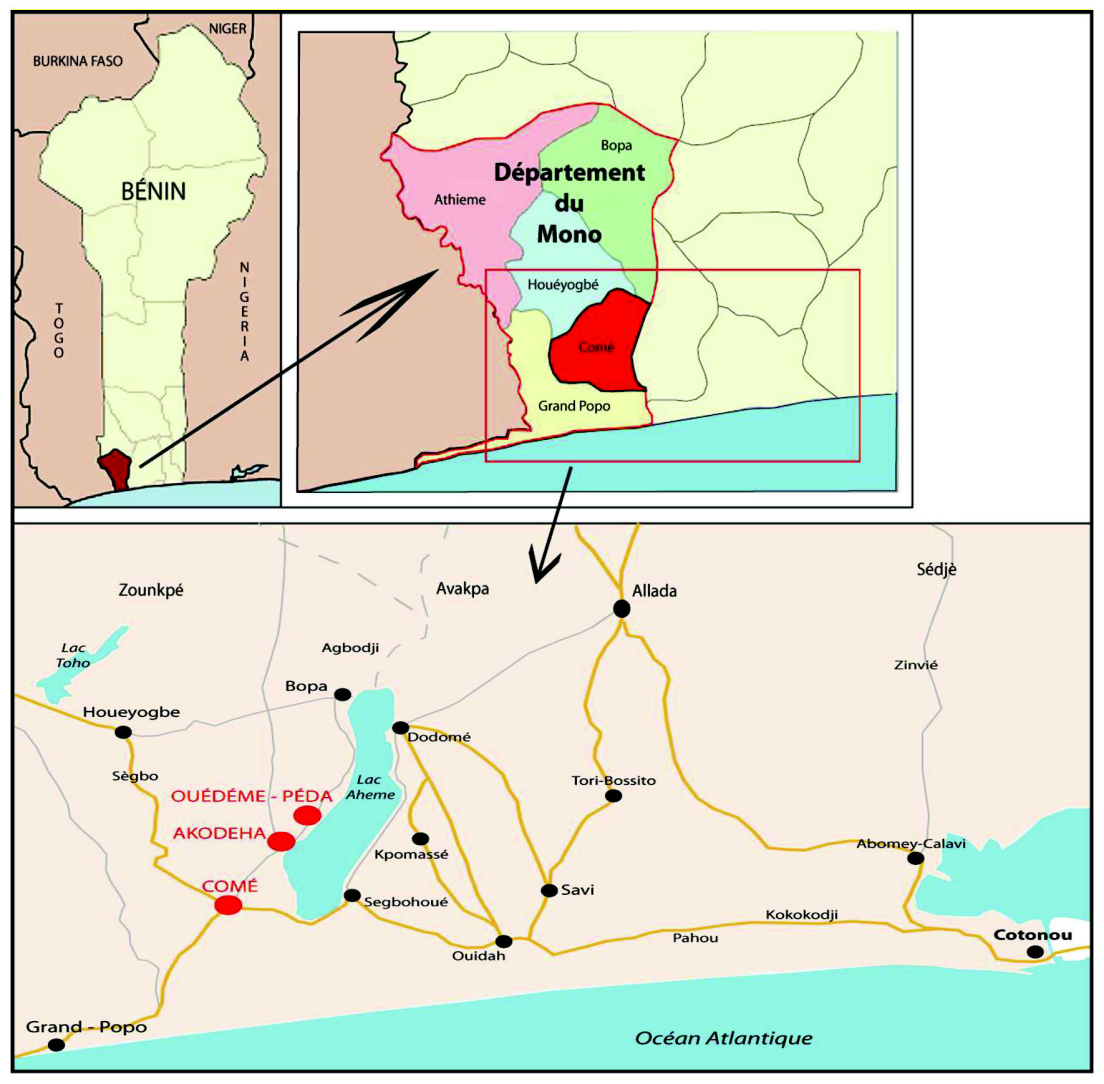
**Figure 1. Descriptive map of the study area (Charlotte Pierrat, UMR216).**

### Study population

The study participants were drawn from the STOPPAM project’s overall cohort of >1000 pregnant women [19,20]. 

### Ethics statement

The study received ethical approval from Institutional Review Boards: the « Comité consultatif de déontologie et d'éthique » of IRD in France and from the « Comité d’éthique de la Faculté des Science de la Santé, Université d’Abomey Calavi »  in Benin. All the participants involved in our study provided their written informed consent to participate in this study; the pregnant women for their follow-up and the caretakers of the minors/children for their follow-up.

### Follow-up of mothers

Pregnant women with a gestational age <24 weeks were enrolled at their first antenatal visit (ANV) after giving written informed consent. They were followed-up monthly until delivery with, on each occasion, a clinical examination, a rapid diagnostic test (RDT, Parascreen, Zephyr Biomedical Systems, Goa, India) to identify plasmodial infections, preparation of a thick blood smear (TBS, for retrospective confirmation of infections), and routine hematological and blood biochemical analyses. Gestational ages were determined by ultrasound as previously described [19]. According to national guidelines, sulfadoxine-pyrimethamine intermittent preventive treatment (SP-IPTp) was administered [21]. In case of clinical symptoms between ANVs, mothers were encouraged to seek care at the maternity clinic. During these unscheduled visits, clinical and biological information were registered as for ANV. Any woman identified as infected by RDT immediately received a treatment regimen of quinine, or of SP if it was the scheduled visit for IPTp.

### Delivery

At delivery, blood samples were collected from the placenta and umbilical cord, blood smear and TBS made from maternal peripheral, placental and cord blood. The newborn had a full clinical examination [14]. The first 218 infants meeting the following criteria: singleton, born in one of the study health centers from an HIV seronegative mother, were enrolled in the study after the parents’ written informed consent.

### Follow-up of infants

Infants had monthly planned visits at the health center. Axillary temperature and hemoglobin concentration (HemoCue®) were measured, and a TBS was prepared. Between monthly visits, home visits were performed to check the infants’ health status, temperature and bednet use. For clinical symptoms between visits, parents were encouraged to seek care the health center. In case of fever (axillary temperature ≥37.5°C) or symptoms suggestive of malaria attack during any visit, an RDT was performed and a TBS prepared. If the RDT was positive, a blood sample was taken, and if febrile the infant was treated with artemisinin-lumefantrine, according to national guidelines. In case of any illness that the health center could not manage, the infant was referred to the district hospital.

### Malaria detection

Plasmodial infection was assessed by RDT in mothers at each scheduled clinic visit and when fever was detected during emergency visits in order to provide appropriate care immediately. RDT were used for immediate diagnosis of infants presenting with fever or other symptoms suggestive of malaria. TBS prepared during both mother and infant follow-ups were processed as described [19]. 

### Statistical analysis

Plasmodial parasitemia was defined as at least one *Plasmodium* parasite present in a TBS or a positive RDT. A malaria attack was defined as any parasitemia with fever (≥37.5°C). Malaria episode duration was estimated at 21 imputations days to avoid counting episodes twice. The timing and number of infections during pregnancy were assessed with reference to peripheral blood parasitemia determined from TBS. Placental infection was analyzed separately from peripheral infections, and was determined by examination of placental impression smears.

Body-mass-index (BMI) was calculated from post-delivery weights. Women were considered underweight for a BMI<18.5 kg/m^2^ and overweight for a BMI≥25 kg/m^2^.

Gestational age was evaluated by ultrasound for 97.4% of the women, and by symphysis-fundus length estimation for the remainder. The first trimester of pregnancy was defined as <93 days of gestation, the second as 94-185 days, and the third >186 days. Premature delivery was defined as <37 weeks, and low birth weight (LBW) as <2500g. Maternal anemia was defined as a hemoglobin concentration <11g/dL [22]. Seasons (dry/rainy) were determined from pluviometric data of southern Benin during the project period. Location of residence was considered “near Lake Ahémé” if located within 0.5 mile of the lake.

The analysis aimed to evaluate the effect of both the timing of occurrence and the number of plasmodial infections during pregnancy on two infant outcomes: parasitemia and malaria attacks during the first year of life. The variable “time of infection” was divided into 3 binary variables, corresponding to pregnancy trimesters. To deal with missing information relative to women not attending first trimester ANV, multiple imputations by chained equation (MICE) [23], base on a Monte-Carlo Markov chain algorithm under missing at random (MAR) hypothesis was used:

 20 multiple sets of simulated values were performed [24]. The variable “number of infections” was divided into 3 classes (None/One/Two or more). These PAM-related variables were studied in univariate analysis for each infant outcome, and entered into multivariate models including placental infection and other covariates with a p value <0.2 in the univariate analysis.

The effect of timing and number of PAM episodes on parasitemia and malaria attacks in infancy was assessed by logistic regression. The effect of PAM on the occurrence of first parasitemia and first malaria attack was assessed by Kaplan-Meier analysis, followed by a semi-parametric Cox regression. The proportional hazards assumption was verified for all variables included in the model. All p values were two-sided, and confidence intervals (CIs) were calculated at the 95% level. Statistical significance was set at p≤0.05. Data analyses were conducted using STATA version 11.0 (Stata Corporation, College Station, Texas, USA).

## Results

### Study population

218 mother-infant pairs were enrolled between November 2008 and April 2010. They had similar characteristics to the other 601 pairs in STOPPAM, except for geographic origin (a higher proportion of children from Akodeha, p=0.01) and parasitological data (more frequent parasitemias in the mothers of the children followed-up, p=0.003 [peripheral] and p<0.001 [placental]). 

194 mother-infant pairs were selected for final analyses (Figure 2), including 181 (93.3%) infants followed for 12 months. The average number of planned visits was 19±5/infant (79% of total planned visits), with no differences between health centers. The general characteristics of these mother-infant pairs are shown in Table 1. 

**Figure:**
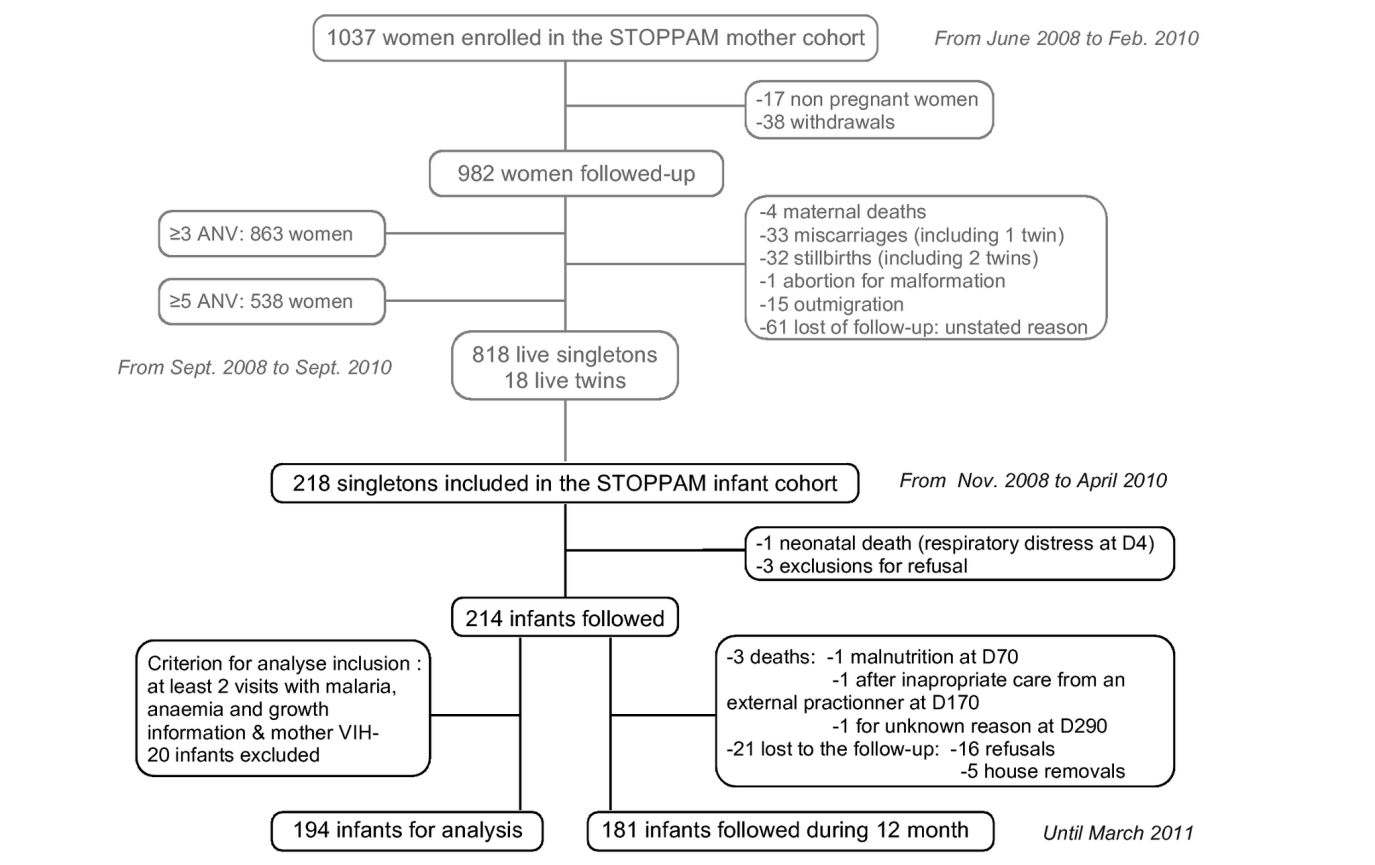
**Figure 2. Flowchart diagram of STOPPAM follow-up: Infants who were absent during ≥4 months, and not seen before their 12 months were considered as lost to follow-up (5 home removal and 16 refusal). For each, the field team checked that it was not due to medical reasons. Mothers of infants lost to follow-up were younger than the others, but all other characteristics were similar.** Four infants died during the follow-up: one perinatal death after a respiratory distress syndrome, and 3 after more than 28 days, due to severe malnutrition and respiratory infection. None of those infants was premature, born with a low birth weight, or had any distinctive characteristics from the others. Twelve infants who poorly took part to the follow-up (less than 2 visits with information on malaria and anaemia) were excluded from the analyses. Because of HIV test shortage, 8 mothers were not tested and their infant was also excluded from the analyses.

**Table 1:** Mothers and offspring baseline characteristics of analytical population.

**Category**	**Characteristic**	**Class**	**All**	**PAM negative^a^**	**PAM positive^b^**	**p**
	Number of mother-infant pairs		194	88	106	
**General Area**	Health centre (%)	Akodeha	41.8	30.7	50.9	0.015
		Come	40.7	50.0	33.0	
		Ouedeme	17.5	19.3	16.0	
	Residence near lake (%)		59.3	50.0	67.0	0.017
	Residence area (%)	Rural	27.8	22.7	32.1	NS
		Semi-rural	72.2	77.3	67.9	
**Mother Characteristics**	Age (years ± SD)		26.5 ± 6.0	27.7 ± 5.8	25.6 ± 5.9	0.020
	Gravidity (%)	Primigravidae	18.6	13.6	22.6	NS
		Secundigravidae	21.7	22.7	20.8	
		Multigravidae	59.8	63.6	56.6	
	Ethnic group (%)	Adja	16.5	17.1	16.0	NS
		Fon	8.3	9.1	7.6	
		Pedah	30.4	25.0	34.9	
		Sahoue	15.5	19.3	12.3	
		Watchi	20.1	20.5	19.8	
		Other	9.3	9.1	9.4	
	Education (%)	No education	54.1	53.4	54.7	NS
		Primary	30.9	28.4	33.0	
		Secondary	15.0	18.2	12.3	
	BMI at delivery (%)	Underweight	8.3	5.7	10.4	0.040
		Normal	77.8	73.9	81.1	
		Overweight	13.9	20.5	10.4	
	Gestational age at inclusion (weeks ±SD)		16.8 ± 4.7	17.5 ± 4.2	16.3 ± 5.0	NS
	ANV number (%)	≤4 ANV	37.6	36.4	38.7	NS
		>4 ANV	62.4	63.6	61.3	
	Anaemia during pregnancy (%) ^c^		89.2	84.1	93.4	0.030
	Anaemia at delivery (%)		45.9	37.0	53.0	0.032
**Offspring Characteristics**	Gender (%)	Female	46.4	46.6	46.2	NS
		Male	53.6	53.4	53.8	
	Birth season (%)	Dry	60.3	62.5	41.5	NS
		Humid	39.7	37.5	58.5	
	GA at delivery (Weeks ± SD)		39.7 ± 1.7	39.9 ± 1.4	39.5 ± 1.9	NS
	Low Birth weight (%)		9.8	6.8	12.3	NS
	Weight (g ± SD)		3040 ± 430	3100 ± 400	2990 ± 450	0.04

NS: Not Significant

a Women with no parasitemia (peripheral or placental) detected during pregnancy

b Women with a least one parasitemia (peripheral or placental) detected during pregnancy

c Women with a least one visit with anaemia ([Hb] <11g/dL) during pregnancy

During pregnancy, 13 women were infected during the 1^st^ trimester, 34 during the 2^nd^, and 57 during the 3^rd^ trimester. Sixty-four pregnant women experienced at least one infection, and 42 experienced 2 or more. Thirty-one mothers had infections in two different trimesters. Thirty-six had a placental infection. The PAM positive mothers (with at least one peripheral or placental infection during pregnancy) lived closer to the lake, were younger, were more anemic during pregnancy, in particular at delivery, and delivered infants with a lower birth weight (Table 1).

During their first year of life, 63 infants (32%) experienced at least one infection, 50 (26%) at least one malaria attack, 17 (8%) at least 2, and 4 infants (2%) at least 3 malaria attacks. Infants with no *P. falciparum* infections were followed for the same time as the others. Fifty-six percent of infants who experienced a malaria attack were born to a mother who had had at least one infection during the last trimester of pregnancy vs 31% of infants without a malaria attack (p=0.002).

### Effect of PAM on the occurrence of infant *parasitological and clinical* malaria

Univariate analysis of the risk of parasitemia or malaria attack during infancy was performed first, followed by multivariate logistic regression. Taking the timing of PAM as the main predictor variable (Table 2), both univariate and multivariate analyses showed that only third trimester infections were associated with parasitemia (AOR 4.2, 95% CI [1.6-10.5]) or malaria attacks (AOR 4.6 [1.7-12.5]) in infants. After adjustment, other factors associated with parasitemia and malaria attacks included use of bednets (protective, with AORs of 0.2 [0.06-0.4] and 0.3 [0.1–0.8], respectively) or birth season (AOR 0.5 [0.3-0.9] and 0.2 [0.06–0.4], respectively). Residence near Lake Ahémé was related to malaria attacks only (AOR 3.8 [1.4-10.8]). Interestingly, placental infection was related neither to parasitemia nor to malaria attacks in infancy.

**Table 2:** Effect of timing of PAM on infant parasitemia and clinical malaria - logistic regression.

Characteristic		n (%)	**Infant parasitemia**					**Infant clinical malaria**			
			Crude Odds ratio	p-value	Odds ratio [95%CI]	p-value		Crude Odds ratio	p-value	Odds ratio [95%CI]	p-value
PAM 1^st^ trimester ^a^	No	(73.1) ^b^	Ref.		Ref.			Ref.		Ref.	
	Yes	(26.9) ^b^	1.33 [0.36-4.94]	0.663	1.12 [0.23-5.45]	0.887		1.19 [0.35-4.08]	0.775	0.83 [0.13-5.18]	0.837
PAM 2^nd^ trimester ^a^	No	142 (73.2)	Ref.		Ref.			Ref.		Ref.	
	Yes	52 (26.8)	1.14 [0.58-2.23]	0.700	0.87 [0.35-2.09]	0.751		1.24 [0.61-2.52]	0.554	0.81 [0.31-2.12]	0.663
PAM 3^rd^ trimester ^a^	No	121 (62.4)	Ref.		Ref.			Ref.		Ref.	
	Yes	73 (37.6)	2.77 [1.49-5.15]	0.001	4.16 [1.64-10.54]	0.003		2.8 [1.45-5.42]	0.002	4.61 [1.70-12.45]	0.003
Placental infection	No	154 (81.1)	Ref.		Ref.			Ref.		Ref.	
	Yes	36 (19.0)	1.58 [0.75-3.32]	0.231	0.72 [0.25-2.11]	0.553		1.53 [0.70-3.34]	0.290	0.59 [0.18-1.88]	0.370
Mother age (years)	<25	94 (48.5)	1.39 [0.73-2.64]	0.316	1.73 [0.78-3.86]	0.180		1.64 [0.83-3.28]	0.157	2.35 [0.97-5.73]	0.059
	≥25 and <35	78 (40.2)	Ref.		Ref.			Ref.		Ref.	
	≥35	22 (11.3)	1.42 [0.53-3.77]	0.483	1.06 [0.29-3.83]	0.927		1.39 [0.48-4.01]	0.545	1.06 [0.26-4.31]	0.931
Residence close to the lake	No	79 (40.7)	Ref.		Ref.			Ref.		Ref.	
	Yes	115 (59.3)	3.91 [1.94-7.86]	<10^-3^	1.90 [0.79-4.57]	0.154		6.14 [2.59-14.56]	<10^-3^	3.83 [1.36-10.83]	0.011
Bed net utilization ^c^	Partially	37 (20.1)	Ref.		Ref.			Ref.		Ref.	
	Yes	147 (79.9)	0.48 [0.12-1.98]	<10^-3^	0.16 [0.06-0.42]	<10^-3^		0.20 [0.09-0.44]	<10^-3^	0.32 [0.12-0.82]	0.018
Birth season	Dry	117 (60.3)	Ref.		Ref.			Ref.		Ref.	
	Humid	77 (39.7)	0.49 [0.26-0.93]	0.030	0.42 [0.18-0.98]	0.046		0.33 [0.16-0.70]	0.004	0.29 [0.11-0.75]	0.011

a Peripheral parasitemia

b Data obtained with logistic imputation

c As every infants used a bed net at least once, there is no category “No bed net use”. To avoid power lost for multivariate analysis, the category “Unknown” has been added when information about bed net utilization was missing (category not shown)

Taking the number of PAM episodes as the predictor variable showed only borderline associations for women experiencing ≥2 episodes (compared to 0), with an AOR of 2.45 (0.90-6.64) (p = 0.08) for infant parasitemia, and 2.15 (0.75-6.14) (p = 0.15) for malaria attacks. Other variables remained associated with parasitemia and malaria attacks: use of bed nets, birth season and residence near the lake.

### Effect of PAM on the time to first parasitemia and first malaria attack

The Kaplan Meier curves, according to PAM timing and the number of PAM episodes, are presented in Figure 3.A. Infant survival probabilities of not presenting with a first malaria infection within 12 months were, respectively, 0.5 (0.4-0.6) and 0.8 (0.7-0.8) according to the presence or absence of PAM during the third trimester. The probabilities of not presenting with a first malaria attack within 12 months were 0.6 (0.5-0.7) and 0.8 (0.7-0.9). Infants born from mothers with PAM in the third trimester had a first parasitemia on average after 285 days (362, 18-390) (respectively median, min and max) whereas infants born from non-infected mothers were first infected on average after 321 days (365, 64-449). Infants born to mothers with a PAM episode during the third trimester had a higher probability of first infections and first malaria attacks than infants born to non-infected mothers (Log-Rank tests, both p≤0.004).

**Figure:**
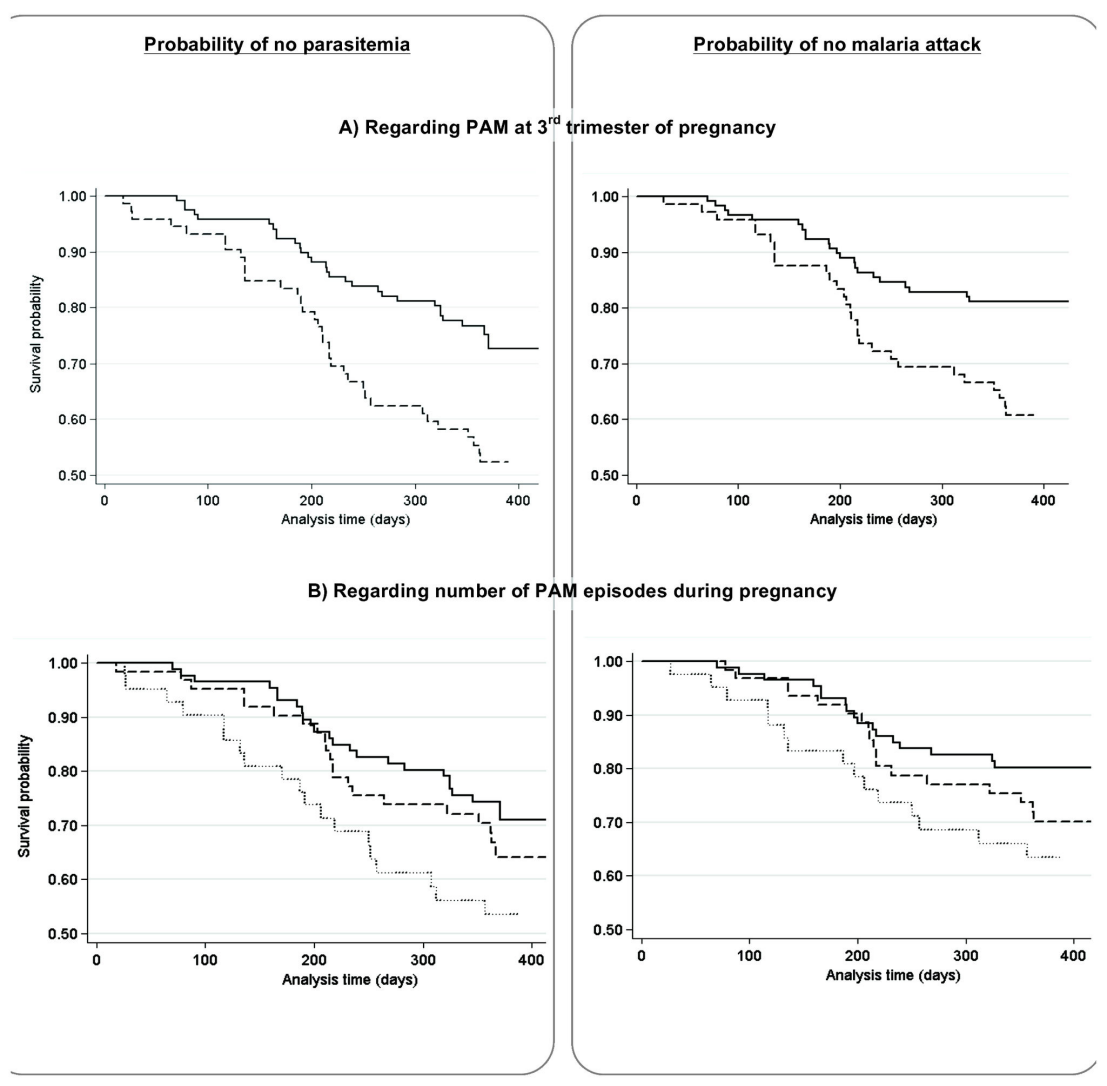
**Figure 3. Kaplan-Meier analysis of the probability of not presenting with parasitemia (left panels) or malaria (right panels) from birth to 12 months of age in infants born to mothers (A) with (dashed line) or without (solid line) malaria infection during the 3^rd^ trimester of pregnancy or (panel B) with no (solid line), one (dashed line), or two or more (dotted line) malaria infection during follow-up.**

When the number of PAM episodes was taken as the predictor variable instead of timing, infant survival probabilities of not presenting with a first infection within 12 months were respectively 0.5 (0.4-0.7), 0.7 (0.5-0.8) and 0.7 (0.6-0.8) according to the presence of ≥2, 1 or no PAM episodes during pregnancy. The probabilities of not presenting with a first malaria attack within 12 months were 0.7 (0.5-0.8), 0.7 (0.6-0.8) and 0.8 (0.7-0.9) for ≥2, 1 or no PAM episodes respectively (Figure 3.B). Infants born to mothers having had ≥2 PAM episodes had a higher probability of a first infection and a first malaria attack than infants born to non-infected mothers during pregnancy (Log-Rank tests, both p≤0.03)

Cox multivariate analysis: All covariates respected the proportional hazard assumption. We found very similar results to the logistic regression, with a strongly significant role of PAM episodes in the third trimester of pregnancy in increasing the risk of parasitemia (AHR 3.0 [1.6-5.5]) or malaria attacks (AHR 3.2 [1.6–6.4]) in infancy (Table 3). Placental malaria had no observable effect. Bed net use and birth season had an impact on both outcomes, and maternal age was related to malaria attacks.

**Table 3:** Effect of timing of PAM on first infant parasitemia and first clinical malaria – Cox regression.

Characteristic		**Infant parasitemia**					**Infant clinical malaria**			
		Crude Hazard ratio	p-value	Hazard ratio [95%CI]	p-value		Crude Hazard ratio	p-value	Hazard ratio [95%CI]	p-value
PAM 1^st^ trimester ^a^	No	Ref.		Ref.			Ref.		Ref.	
	Yes	1.30 [0.57-2.92]	0.527	1.00 [0.42-2.39]	0.999		1.44 [0.49-4.19]	0.497	0.97 [0.32-2.92]	0.950
PAM 2^nd^ trimester ^a^	No	Ref.		Ref.			Ref.		Ref.	
	Yes	1.27 [0.73-2.19]	0.395	1.14 [0.62-2.12]	0.675		1.35 [0.74-2.47]	0.332	1.15 [0.58-2.28]	0.697
PAM 3^rd^ trimester ^a^	No	Ref.		Ref.			Ref.		Ref.	
	Yes	2.23 [1.36-3.65]	0.002	2.95 [1.58-5.50]	0.001		2.26 [1.29-3.96]	0.004	3.19 [1.59-6.38]	0.001
Placental infection	No	Ref.		Ref.			Ref.		Ref.	
	Yes	1.41 [0.79-2.52]	0.243	0.68 [0.34-1.38]	0.291		1.33 [0.69-2.54]	0.395	0.60 [0.28-1.32]	0.205
Mother age (years)	<25	1.35 [0.80-2.30]	0.264	1.68 [0.94-3.00]	0.079		1.51 [0.84-2.74]	0.172	2.05 [1.05-3.98]	0.034
	≥25 and <35	Ref.		Ref.			Ref.		Ref.	
	≥35	1.28 [0.58-2.83]	0.534	1.39 [0.59-3.29]	0.451		1.30 [0.52-3.23]	0.578	1.47 [0.54-4.01]	0.456
Residence close to the lake	No	Ref.		Ref.			Ref.		Ref.	
	Yes	3.24 [1.76-5.97]	<10-3	1.89 [0.94-3.79]	0.074		5.25 [2.36-11.68]	<10-3	3.45 [1.42-8.37]	0.006
Bed net utilization ^b^	Partially	Ref.		Ref.			Ref.		Ref.	
	Yes	0.23 [0.14-0.39]	<10-3	0.29 [0.16-0.52]	<10-3		0.26 [0.14-0.46]	<10-3	0.40 [0.21-0.76]	0.005
Birth season	Dry	Ref.		Ref.			Ref.		Ref.	
	Humid	0.53 [0.31-0.92]	0.025	0.46 [0.24-0.88]	0.019		0.37 [0.19-0.73]	0.004	0.35 [0.17-0.76]	0.007

a Peripheral parasitemia

b As every infants used a bed net at least once, there is no category “No bed net use”. To avoid power lost for multivariate analysis, the category “Unknown” had been add when information about bed net utilization was missing (category not shown)

The effect of the number of PAM episodes on first parasitemia and malaria attack was also significant with a 2.3- ([1.2 - 4.8], p=0.02) and a 2.2- ([1.0 - 4.9], p=0.05) fold increase in the risk for these two outcomes for mothers who experienced a malaria infection twice or more.

## Discussion

Previous studies demonstrated that infants born to mothers with placental infection at delivery were at increased risk of parasitemia or malaria attacks in the first year of life, but in none of the published studies were the women followed during pregnancy, and hence the analyses could not take account of maternal infection histories prior to delivery. The STOPPAM project, of which the study described here was a part, included longitudinal follow-up as an integral component. Importantly, almost all participating women (97.4%) had an early ultrasound examination, the reference method, resulting in very accurate determination of gestational ages [25,26]. Coupled with the latter, regular monitoring for the presence of parasites in peripheral blood allowed precise evaluations of the timing of infections during pregnancy, and therefore to assess whether variations in such timing resulted in differential impacts on infant health. Our results show for the first time that infections of the mother occurring in the third trimester, rather than those occurring earlier or those identified at delivery, were strongly associated with an increased susceptibility both to parasitemia and to malaria attacks during infancy.

From a biological point of view, the increased susceptibility to malaria of the infant is thought to result from *in utero* exposure to soluble parasite-derived antigens, leading to altered immune responses – frequently characterized as ‘tolerance’ – to these antigens that are detectable at birth but also later in life [15,27-30]. Besides this specific malaria context, a growing body of evidence points to susceptibility to disease in childhood being largely determined *in utero* or early in infancy following exposure during pregnancy to environmental immunotoxicants [31], urban air pollution [32], infectious agents [33], or undernutrition [34,35]. The data we present here on the effects of intra-uterine exposure on infants’ susceptibility to malaria are clearly consistent with these findings.

Our results indicate a major role of maternal parasitemia present late in pregnancy, emphasized by the association with a more than 4-fold increased risk of the child suffering a malaria attack during the first year of life. This is consistent with our own previous reports of the deleterious effects of infection with *P. falciparum* during the third trimester of pregnancy, characterized by poor birth outcomes such as low birth weight or maternal anemia at delivery [20,36]. Strikingly, our data also show that multiple (≥2) maternal parasitemic events during pregnancy were significantly associated with a 2-fold increased risk of malaria attack in the infant. Notably, maternal infections occurring earlier in pregnancy were not associated with any increased risk for the child. The second trimester is the period when the two doses of SP-IPTp are generally given (on average between 4 and 6 months’ gestational age in the STOPPAM study) [37]. In areas like our study site, where the prevalence of parasite mutations associated with resistance to SP is high, IPT administration may not completely clear parasites, but will reduce parasite density below the detection level of TBS (N. Ndam, personal communication). Given that the antiparasitic effect of SP persists for several weeks, parasitemia may thus remain undetected for this period of time, as suggested by the high number of *P. falciparum* infections detected long after the last dose of IPTp was given [18]. The first trimester is also considered as crucial with respect to consequences for the newborn, in particular LBW [20,36]. However, a significant number of women were not followed prior to the fourth month of pregnancy, so their early infection history is unknown. The relevant missing data was taken into account by using a multiple imputation technique in the statistical analysis, but the information available from the first trimester of pregnancy is comparatively much smaller than for the later trimesters, with a consequent lack of power. Nevertheless, the differences between the first-second and third trimesters of gestation are large, probably reflecting the major role played by late maternal infections. This is consistent with the fact that the fetal immune system develops throughout the third trimester, with a critical T-cell repertoire generation through birth [38]. 

In marked contrast with previous studies, and although parasitemia or malaria attacks were more common in infants born from infected placentas, the association did not reach significance. Until now, placental malaria has been considered a surrogate for infections arising earlier, and a 10-fold increased risk of placental infection in women parasitized after 7 months of pregnancy has been reported [39]. The recent implementation of IPTp in all sub-Saharan African countries has undoubtedly improved anti-malarial protection, clearing most placentas of parasites, such that placental malaria at delivery no longer reflects longer-lasting infections as it did in ‘pre-IPTp’ studies. Although, in the STOPPAM study, comparatively early administration of the last dose of SP-IPTp did result in an increased prevalence of placental infections [37], the routine monthly surveillance of mothers precluded chronic carriage, a fact confirmed by our genotypic analyses showing that a high proportion of placental infections were actually acquired 4 weeks or less before delivery [40]. Given the relatively small size of our sample and the resulting lack of power of the statistical analysis, it is interesting to note that the two studies conducted after IPTp implementation showed only border-line associations between placental malaria and the onset of first parasitemias [14], although the numbers of children followed were large (over 450, more than twice our sample size).

This study is one of the few where clinical symptoms were systematically recorded during the children’s follow-up. Probably reflecting the immaturity of infants’ immune responses, nearly 80% of parasitemias were associated with fever. Interestingly, we found very similar results using both variables as main endpoints (parasitemia and clinical symptoms), with increased risks between 4 and 5-fold for PAM during the third trimester, and between 2 and 3-fold for a number of PAM episodes ≥2. This finding is partly explained by the high proportion of symptomatic children, but also confirms the importance of the number of gestational infections in terms of consequences for the baby.

This study was primarily designed for immunological investigations of infants’ first infections, rather than for epidemiological purposes, and thus has limitations. First, the mother-infant pairs selected for follow-up may not be fully representative of the overall population of STOPPAM women and their offspring. Although most variables did not differ, the rate of placental infection was higher in the 218 pairs than in the remaining 601 (19.7% vs 7.5%), which may be explained by a higher rate of recruitment in the second and third trimesters of the year, at STOPPAM’s mid-term, when malaria transmission was highest. Nevertheless, as our main purpose was to study the relationship between PAM and parasitemia in infancy, representativeness of the sample is not obligatory, and a potential selection bias towards more heavily infected mothers, although unlikely, should not have modified the associations we observed.

Second, more frequent parasitemia during pregnancy suggests greater exposure to transmission, with infants sharing the same environment equally exposed, giving a possible alternative explanation to the associations we found. This emphasizes the importance of carefully assessing malaria exposure in such studies [41]. No entomological study could be performed at the time of the infant follow-up, but study villages were located in distinct areas as regards their proximity to Lake Ahémé, and we used these data as a surrogate of vector exposure. Although not a precise measure of entomological transmission at the household level, it was closely related to the occurrence of parasitemia during pregnancy, and was introduced in the regression models for adjustment, along with birth season. 

Third, RDTs were used to diagnose parasitemias during pregnancy. The sensitivity and specificity of this method is roughly comparable to thick smear examination. By identifying circulating plasmodial antigens, RDTs may detect parasites sequestered in the placenta, but more sensitive PCR methods detect submicroscopic parasitemias. RDTs do not detect such small parasite loads that may also play a role in the susceptibility of infants to malaria.

In conclusion, the present study showed that both maternal parasitemias during the third trimester, and the number of malaria infections during pregnancy were associated with an increased risk of infections and malaria attacks during the first year of life. This highlights the importance of improving malaria prevention strategies during pregnancy to optimally protect the infant. In this respect, the recent recommendations of the WHO to give SP-IPTp at each scheduled antenatal visit with an advised number of four visits, will probably facilitate the systematic use of a third dose of IPTp. This should help to decrease infants’ susceptibility to malaria by shifting the last IPTp dose into the latter part of pregnancy. The development of a PAM-specific vaccine, obviating the need for IPTp, should similarly lower the burden of malaria in early life.
